# Avascular Necrosis and Minimal Trauma Fractures in Telomere Biology Disorders

**DOI:** 10.1111/cge.70038

**Published:** 2025-08-05

**Authors:** Arman M. Niknafs, Neelam Giri, Marena R. Niewisch, Sharon A. Savage

**Affiliations:** ^1^ Clinical Genetics Branch, Division of Cancer Genetics and Epidemiology National Cancer Institute Bethesda Maryland USA

**Keywords:** avascular necrosis, bone health, dyskeratosis congenita, fracture, telomere, telomere biology disorder

## Abstract

Avascular necrosis (AVN) and minimal trauma fractures (MTF) cause significant morbidity in patients with telomere biology disorders (TBDs). TBDs are associated with very high risks of bone marrow failure, pulmonary fibrosis, cancer, and many other complications due to pathogenic germline variants in genes essential for telomere function and maintenance. To understand the extent to which AVN and MTF occur in TBDs and identify areas requiring more research in the role of telomeres in bone biology. We assessed the occurrence of AVN and MTF in 233 patients with TBDs. An age, gender, and gene‐matched TBD patient control group was used to assess associations between AVN/MTF and clinical characteristics. Forty‐two (18%) patients with TBD developed at least one AVN and/or MTF event with 19 patients experiencing their first event in childhood. AVN and MTF were most common in patients with autosomal or X‐linked recessive, or heterozygous *TINF2* disease (19/36 AVN and 17/19 MTF). Androgen and corticosteroid use were more common in patients with AVN compared with matched patient controls (41.2% vs. 16.3%, *p* < 0.05 and 41.2% vs. 14%, *p* < 0.01, respectively); however, 57.1% of patients experienced AVN and/or MTF events in the absence of androgen or corticosteroid use. Severe bone marrow failure and hematopoietic cell transplantation history were significantly more common in MTF patients than in controls (44.2% and 30.2% respectively, *p* < 0.05). There were no statistically significant associations between low bone mineral density or vitamin D deficiency and AVN or MTF. AVN and MTFs are common, debilitating complications in TBDs and frequently occur independently of androgen or corticosteroid use. Our results underscore the need for disease‐specific translational studies as well as improved prevention and therapeutic options for patients with TBDs.

**Trial Registration:** ClinicalTrials.gov identifier: NCT00027274

## Graphical abstract

Avascular Necrosis (AVN) and Minimal Trauma Fractures (MTF) cause significant morbidity in people with Telomere Biology Disorders (TBDs). They occur more frequently in young patients and in those with autosomal recessive (AR) or X‐linked recessive (XLR) than in autosomal dominant (AD) genotypes.

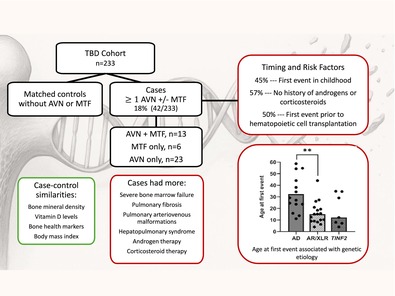

## 1 Introduction

Telomere biology disorders (TBDs) are associated with very high risks of bone marrow failure, pulmonary fibrosis, head and neck squamous cell carcinoma, myelodysplastic syndrome, acute myeloid leukemia, stenosis of the esophagus, urethra, and lacrimal ducts, liver fibrosis, avascular necrosis (AVN) of the femoral or humeral head(s), and many other complications [1, 2, 3, 4]. Dyskeratosis congenita (DC), the first described TBD, includes these features as well as the mucocutaneous triad of nail dysplasia, oral leukoplakia, and abnormal skin pigmentation. Individuals with TBDs have short and/or dysfunctional telomeres due to pathogenic germline variants in essential telomere maintenance genes. Variants in at least 18 different genes are associated with TBDs with multiple modes of inheritance including X‐linked recessive (*DKC1*), autosomal dominant (AD: *ACD*, *NAF1*, *NHP2*, *NOP10*, *PARN*, *POT1*, *RPA1*, *RTEL1*, *TERC*, *TERT*, *TINF2*, *ZCCHC8*), and autosomal recessive (AR: *ACD*, *CTC1*, *DCLRE1B*, *NOP10*, *PARN*, *POLA2*, *POT1*, *RTEL1*, *STN1*, *TERT*, *WRAP53*). Heterozygous *TINF2* variants often occur de novo [2, 3, 5, 6].

AVN or nontraumatic osteonecrosis is a progressive degenerative pattern of bone resorption and formation most commonly impacting the hip joint and resulting in sclerosis and subchondral collapse of the bone [7, 8, 9, 10]. It is generally considered to result from decreased blood flow to subchondral bone, with a multifactorial etiology including vascular interruption, microvascular thrombotic disease, and intraosseous extravascular compression secondary to adipocyte hypertrophy [11].

Minimal trauma fracture (MTF) generally refers to bone fragility or low‐trauma fractures occurring in the setting of low bone mineral density (BMD) and/or osteoporosis, which primarily affects older adults [12]. MTFs in older adults are caused by an imbalance of bone remodeling where bone resorption outpaces bone formation in post‐menopausal women, and conditions including malignancy, hyperparathyroidism, osteomalacia secondary to vitamin D deficiency, and adynamic bone disease [13]. Low BMD and MTFs in children are associated with rare genetic disorders (e.g., osteogenesis imperfecta) or secondary causes such as nutritional deficiencies, endocrine dysfunction, and longstanding glucocorticoid use [14, 15, 16].

Children and young adults with TBDs can develop AVN of the femoral and/or humoral head(s) which often requires joint replacement [17, 18, 19, 20, 21, 22, 23, 24, 25, 26, 27, 28, 29, 30]. MTF and poor bone healing are characteristic of Coats plus, a TBD primarily caused by biallelic *CTC1* variants [31]. AVN and MTFs have been attributed to androgen or corticosteroid use in some patients with TBDs, but the extent to which therapy contributes to these complications is unknown [4, 32]. Associations between AVN or MTF and low BMD in TBDs are unclear, but one study of BMD in patients with DC/TBD found eight of 30 patients (27%) had low BMD (defined as Z‐score ≤ −2) [33].

We sought to understand the extent to which AVN and MTF occur in TBDs and identify areas requiring more research in the role of telomeres in bone biology. In this retrospective cohort study, we assessed the prevalence of AVN and MTFs in 233 patients with TBDs and determined their association(s) with genotype and other clinical manifestations.

## 2 Materials and Methods

### 2.1 Study Participants

Individuals in this study were enrolled in the National Cancer Institute's (NCI) Institutional Review Board‐approved Inherited Bone Marrow Failure Syndromes (IBMFS) protocol [34]. All participants or their legal guardians provided written informed consent in accordance with Health and Human Services regulation 45 CFR 46. The study opened on November 28, 2001, and the current analyses include data collected through May 31, 2022. Study participants shared medical records and completed detailed questionnaires. A subset of 93 patients underwent comprehensive medical evaluations at the National Institutes of Health Clinical Center in Bethesda, MD [4].

Clinical diagnosis of a TBD was confirmed by germline genetic testing and/or telomere length measurement in peripheral blood lymphocytes via flow cytometry with fluorescent in situ hybridization [35]. Questionnaires and medical records were reviewed in detail and included diagnostic imaging, TBD manifestations, medications, and social history. Laboratory measures of bone and cardiovascular health assessed included erythrocyte sedimentation rate, C‐reactive protein, low density lipoprotein (LDL), high density lipoprotein (HDL), triglycerides, total cholesterol, creatinine, calcium, phosphorous, parathyroid hormone, vitamin D (25‐(OH)D), alkaline phosphatase, and thyroid studies (T3, T4, and thyroid stimulating hormone [TSH]). All relevant laboratory values represented were sourced from the 5‐year period prior to the date of AVN or MTF.

MTFs were defined as radiologically confirmed fractures that occurred spontaneously or following a low‐energy or low‐impact event during routine activities (e.g., getting in a vehicle, or a normal childhood activity such as tripping on the playground). One patient with osteonecrosis and fracture of the mandible following radiation therapy for cancer was excluded from this analysis.

### 2.2 Statistical Analyses

Stratified randomization was performed to create a control group of patients with TBD and who had no history of AVN or MTF to allow for genotype and phenotype comparisons with those with AVN and/or MTF (Figure S1). The two groups were matched 1:1 based on age, sex, affected gene, and pattern of inheritance (autosomal dominant or recessive, X‐linked recessive, *TINF2*, or unknown) [4]. We used the two‐sided Student's *T*‐test and Fisher's exact test to compare parameters from groups affected by bony events (AVN, MTF, or AVN and MTF) to the matched patient controls. A two‐way analysis of variance was completed for telomere length and lipid values between groups. Statistical analysis was performed using RStudio (version 2023.03.1446) and PRISM (version 9.5.0 (525)).

## 3 Results

### 3.1 Participant Characteristics

**FIGURE 1:**
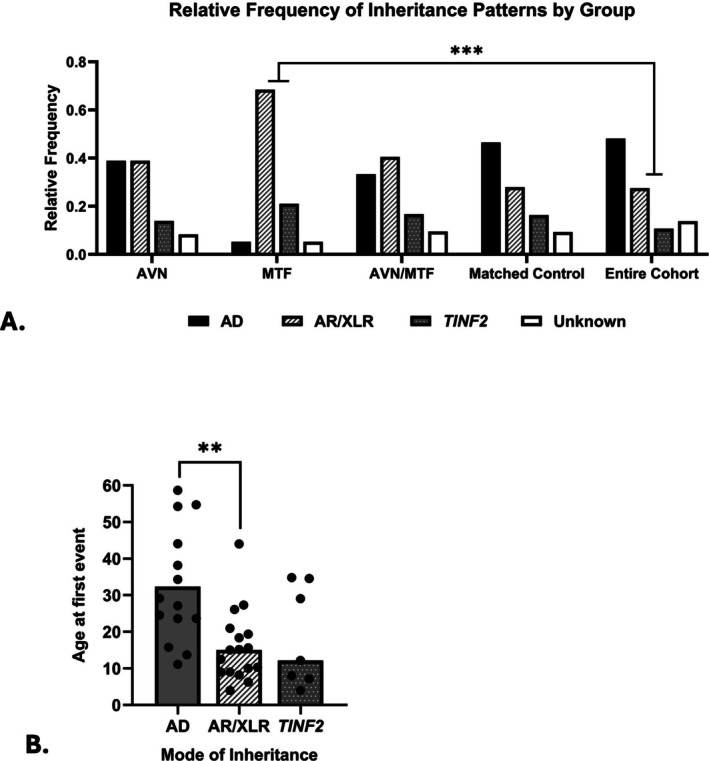
Occurrence of avascular necrosis (AVN) and/or minimal trauma fracture (MTF) by genetic etiology. (A) Age at first event (AVN or MTF) by mode of inheritance; (B) frequency of AVN and/or MTF by patient groups. Individuals with heterozygous variants in *TINF2* were considered separately, as described in the methods. ** indicates *p* < 0.01 and *** indicates *p* < 0.001. AD, autosomal dominant; AR, autosomal recessive, XLR, X‐linked recessive. [Colour figure can be viewed at wileyonlinelibrary.com]

The characteristics of study participants are shown in Table 1. Forty‐two (18%) of 233 patients with TBDs had a radiologically confirmed diagnosis of AVN and/or MTF. Twenty‐three had AVN alone, six had MTF alone, and 13 had both AVN and MTF (AVN + MTF) (Figure S1). Thirty‐eight of the 42 patients (90%) with AVN and/or MTF had known pathogenic germline variant(s) in TBD‐associated genes. AD (non‐*TINF2*) inheritance was noted in 38.9% of patients with AVN compared with 48.1% in the entire cohort (odds ratio [OR] 0.688, 95% confidence interval [CI] 0.31–1.48, *p* = 0.3705, Figure 1A). Compared with patients in the entire cohort, those who experienced MTFs were significantly more likely to have non‐AD TBD (OR 13.63, 95% CI 3.11–124.6, *p* < 0.001, Figure 1A). Nineteen of the 42 (45.2%) patients experienced their first AVN or MTF in childhood (Figure 1B). Patients with AR or XLR TBD experienced their first AVN or MTF at a significantly younger age than patients with AD disease (mean difference = 16.4 years, 95% CI 7.00–25.82, *p* = 0.0013). There was a borderline, non‐statistically significant difference in the age of the first event between patients with *TINF2* disease compared with AD inheritance of PGVs in other genes (mean difference = 13.6 years, 95% CI 0.74–28.40, *p* = 0.061).

**TABLE 1:** Characteristics of study participants.

	AVN	MTF	AVN and/or MTF	Matched control patients	Entire Cohort
Participant Count^a^	36	19	42	43	233
Median age (range)	23.6 (6.3–58.7)	12.2 (4.0–44.0)	21.0 (6.3–58.7)	23.5 (2.9–55.3)	29.6 (1.3–82.2)
Gender					
Female	12	6	15	18	87
Male	24	13	27	25	146
Median Telomere Length *Z*‐Score (range)	−4.16 (−7.04 to −1.86)	−5.19 (−7.15 to −2.42)	−4.28 (−7.15 to −1.86)	−3.43 (−7.01 to −0.04)	−3.51 (−7.94 to +0.87)
TBD Subtype					
Coats plus	2	2	2	—	3
Dyskeratosis congenita	29	15	35	39	201
Hoyeraal Hreidarsson	4	2	4	2	23
Revesz	1	—	1	2	6
Inheritance Pattern					
AD	14	1	14	20	112
AR/XLR	14	13	17	12	64
*TINF2* ^b^	5	4	7	7	25
Unknown	3	1	4	4	32
Affected Gene					
*TERT*	9 ad, 1 AR	1	9 AD, 1 AR	10 ad	48 ad, 1 AR
*RTEL1*	2 ad, 2 AR	2 AR	2 AD, 2 AR	5 ad, 2 AR	29 ad, 15 AR
*DKC1*	6	5	7	5	33
*TERC*	3	—	3	5	30
*TINF2*	5	4	7	7	25
*PARN*	1 AR	1 AR	2 AR	2 AR	4 ad, 4 AR
*CTC1*	3 AR	4 AR	4 AR	1 AR	6 AR
*WRAP53*	1 AR	1 AR	1 AR	2 AR	3 AR
*ACD*	—	—	—	—	2 AD, 1 AR
Unknown	3	1	4	4	32
Low BMD Score^d^ (Total number DEXA scans)	5 (19)	6 (14)	8 (25)	2 (16)	
Severe BMF (%)	26 (72.2)^c^	17 (89.5)^c^	32 (76.2)	19 (44.2)	113 (48.5)
HCT (%)	14 (38.9)	12 (63.2)^c^	20 (47.6)	13 (30.2)	70 (30.0)
Androgen use within 5 years of event (%)	15 (41.2)^c^	8 (42.1)	18 (42.9)^c^	7 (16.3)	
Median duration in years of androgen use (number with defined dose history)	2.34 (13)	2.34 (8)	2.21 (16)	1.86 (6)	
Corticosteroid Use within 5 years of Event (%)^e^	15 (41.2)^c^	4 (21.1)	15 (35.7)	6 (14.0)	

Abbreviations: AD, autosomal dominant; AR autosomal recessive; AVN, avascular necrosis; BMD, bone mineral density; DEXA, dual energy X‐ray absorptiometry scan; HCT, hematopoietic cell transplant; MTF, minimal trauma fracture.

^a^ Self‐reported ethnicities were as follows: AVN and/or MTF: 83% white, 17% other or not reported; Matched control patients: 81% white, 19% other or not reported; Entire Cohort (*n* = 233): 73% white, 27% other or not reported.

^b^ *TINF2* variants are heterozygous, frequently occur de novo, and patients usually present with TBD manifestations in childhood, and are thus considered separately.

^c^ Denotes a statistically significant difference between AVN and/or MTF compared to the matched control patients with a *p* value < 0.05.

^d^ Low BMD classified as *Z*‐score ≤ 2 standard deviations below population mean.

^e^ Quantification of the duration of corticosteroid use is not reported because it was confounded by reports of intermittent dosing and incomplete records.

### 3.2 Avascular Necrosis in TBDs

The median age at first AVN was 23.6 years (range 6 to 58) with 12 of the 36 patients experiencing their first AVN in childhood. The femoral head was the most frequent site of AVN affecting 33 of 36 patients, 22 of whom had bilateral femoral head osteonecrosis. The other sites of AVN were the vertebra, humeral head, knee, and wrist; bilateral AVN also occurred in the humeral heads of two patients and knees of one patient (Table S1). Six of the 14 patients who underwent hematopoietic cell transplant (HCT) had AVN prior to receiving HCT. Twelve patients underwent bilateral hip replacement and five had unilateral hip replacement due to AVN‐related complications (median age at first joint replacement 29.9 years, range 16.5–56.1). Other treatment modalities included core decompression (five patients), resurfacing (two patients), and pharmacologic pain management only (three patients). Median time from AVN event to treatment was 0.81 years (range 0.03–2.6).

### 3.3 Minimal Trauma Fractures in TBDs

Thirty‐two MTFs occurred in 19 patients at a median age of 12.2 years (range 4 to 44) with most MTFs occurring in children (*n* = 12). The most common site for MTF was the femoral diaphysis in 11 patients, followed by the humerus, tibia, clavicles, and calcaneus among other bones (Table S1). Fractures were reported to occur in minimal trauma settings such as “throwing a ball” (distal humerus), “raking leaves during yardwork” (humeral shaft), “cross country running” (mid‐shaft tibia), “dancing at summer camp” (tibial plateau), “lifting a 10 pound hand weight” (humerus), “holding an infant” (proximal humeral shaft), and “entering a vehicle” (femoral shaft). Twelve of the 19 patients who experienced MTFs had undergone HCT, but six of those 12 experienced their first fracture prior to HCT.

### 3.4 BMD and Metabolism

DEXA scan reports were available on 25 patients with AVN and/or MTF and 16 matched control patients. No patients received bisphosphonate therapy prior to AVN or MTF. Low BMD was present in eight of 25 (32%) patients with AVN and/or MTF, but this was not statistically significantly different from BMD in the matched control patients (OR 3.21, 95% CI 0.51–35.8, *p* = 0.265) (Table 1). There were no statistically significant differences in BMD in stratified subgroup analyses comparing BMD in patient controls to those with MTF only, AVN only, MTF with or without AVN history, or AVN with or without MTF history (data not shown).

There were no statistically significant differences in body mass index or mean serum 25(OH)D concentrations between patients with MTF only, AVN only, MTF with or without AVN history, AVN with or without MTF history, or to the entire affected cohort when compared with matched control patients. Serum calcium, phosphorous, creatinine, and parathyroid hormone levels among patients with bone events were similar between subgroups and within respective clinical reference ranges (Table S2). One patient with *TINF2* disease received bisphosphate therapy for low BMD after five MTFs but experienced another MTF several months after initiating treatment.

### 3.5 Use of Androgens and Corticosteroids

Twelve of 36 (33%) patients with AVN and four of 19 (21%) patients with MTF did not have a history of prior HCT, androgen, and/or corticosteroid use. Patients treated with androgens for bone marrow failure were more likely to have AVN within 5 years of treatment compared with the matched control patients (OR 3.61, 95% CI 1.16–12.29, *p* = 0.022). There was a suggestion of an association of MTF with androgen therapy (OR 3.64, 95% CI 0.93–15.02, *p* = 0.051) compared with matched control patients. However, there were no statistically significant differences in the duration of androgen therapy between patients who experienced bone events and those who did not. Notably, more than half of the patients with AVN and/or MTF (24/42, 57.1%) had no history of androgen use within 5 years of developing AVN and/or MTF.

Patients with TBDs and a history of corticosteroid use had an increased risk of AVN compared with the matched control patient cohort (OR 4.32, 95% CI 1.33–15.77, *p* = 0.009). No significant differences in corticosteroid use were identified between the MTF patient cohort and the matched control patients (OR 1.63, 95% CI 0.29–8.07, *p* = 0.479).

### 3.6 Comorbidities Associated With AVN or MTF

Patients with MTF were more likely to have severe bone marrow failure than the matched control patients (63.2% vs. 30.2%, OR 3.86, 95% CI 1.11–14.56, *p* = 0.023) and notably half of them experienced their first MTF before HCT. Other TBD complications including pulmonary fibrosis, pulmonary arteriovenous malformation (PAVMs), and hepatopulmonary syndrome occurred at greater frequencies in patients with AVN and/or MTF than the matched control patients. History of prior deep venous thrombosis (*n* = 2), presence of PAI‐1 4G/5G (*n* = 1) or PAI‐1 4G/4G (*n* = 1) polymorphisms, hyperhomocysteinemia (*n* = 3), Factor V Leiden heterozygosity (*n* = 2), elevated von Willebrand factor (*n* = 1), elevated factor VIII (*n* = 2) were identified in eight (22%) individuals with AVN. LDL, total cholesterol, and triglyceride values were significantly higher in patients who experienced AVN compared with the matched control patients; however, this association was not statistically significant after adjusting for androgen therapy, which is known to increase cholesterol and lipid levels [36]. No significant differences in LDL, total cholesterol, or triglyceride values were found between the MTF group versus the matched control patient cohort, and no differences were identified in HDL values between groups (Table S3).

## 4 Discussion

Bone pathologies including AVN, MTF, and poor healing have been primarily reported in patients with the typical childhood onset TBDs, including classic DC and Coats plus [17, 18, 19, 20, 21, 22, 23, 24, 25, 26, 27, 28, 29, 30]. In this study, we found that approximately 20% of patients with TBDs experienced at least one AVN or MTF event across the spectrum of TBD phenotypes, genetic etiologies, and age ranges. Patients with TBDs due to AR/XLR or *TINF2* pathogenic variants were more likely to have their first AVN and/or MTF in childhood and to experience multiple events, consistent with prior studies of earlier onset TBD complications associated with these genotypes [2, 3, 4, 37].

Using age, gender, and genetically matched TBD control patients without AVN or MTF history, we explored associations between AVN and/or MTF and clinical factors. Notably, approximately 35% of patients with TBDs (15/42) had AVN or MTF in the absence of known risk factors such as prior HCT, androgen or corticosteroid use. While patients with a history of androgen or corticosteroid use were more likely to have AVN and/or MTF than matched control patients, many (57%) patients with AVN and/or MTF had no history of androgen exposure. AVN rates after HCT in this study were similar to those in non‐TBD patients undergoing autologous or allogenic HCT (11% in TBDs compared with 2%–4% and 5%–19%, respectively) [38]; suggesting that HCT did not substantially alter the risk of AVN in this cohort.

Notably, we observed no significant difference in low BMD by DEXA scan, low serum 25(OH)D, or other bone turnover markers (PTH, calcium, and phosphorous) between AVN and/or MTF affected and matched control patients. These findings, alongside data demonstrating that approximately 65% of patients with bony events did not receive prolonged corticosteroid therapy, suggest a complex, multifactorial etiology of TBD associated bone pathobiology that could be intensified following exposure to androgens, corticosteroids, and/or HCT.

The occurrence of AVN and/or MTF was at a substantially younger age in patients with TBDs (median 23 years for AVN and 12 years for MTF) than in the general US population where the mean age for femoral head AVN in men and women is 30–65 years and 54–56 years, respectively, and for the first MTF event in postmenopausal women is 65–68 years [9, 39, 40, 41, 42]. AVN pathogenesis is linked to altered blood flow to the bone, leading to a hypoxic and ischemic milieu where bone resorption outpaces formation, eventually culminating in osteonecrosis and subchondral collapse [11]. Three primary pathways are hypothesized to underlie this ischemia: trauma‐induced interruption, intravascular occlusion (e.g., sickle cell disease, atherosclerosis, or coagulation abnormalities), and intraosseous extravascular compression (e.g., due to adipocyte hypertrophy, obesity, steroids, or androgens). Only one study to date has assessed telomere length in non‐TBD patients experiencing AVN compared with healthy controls and found no association [43].

The etiology of AVN and MTF in patients with TBDs is unknown but could stem from an inherent propensity of TBD‐patient derived BMSCs to preferentially undergo fibrosis and adipogenic differentiation, potentially contributing to intraosseous extravascular compression [44]. Impaired osteoblast differentiation and reduced mesenchymal cell numbers were reported in a *Terc* knockout mouse model, suggesting a connection between aberrant telomere biology and bone formation [45]. Additionally, patients with TBDs are prone to developing vascular abnormalities including PAVM, gastrointestinal telangiectasias, and exudative retinopathy. An underlying vascular defect could similarly contribute to the development of AVN in TBDs, but a detailed study is required to test this hypothesis [46, 47, 48, 49, 50, 51]. Vascular mechanisms could similarly contribute to MTFs with abnormal vascularization affecting bone growth and remodeling. Limited stem cell replicative capacity due to apoptosis or cellular senescence induced by extremely short telomeres could contribute to a pro‐inflammatory state affecting bone growth and healing. Future studies of the connections between bone biology and telomere function are required to understand the mechanism(s) contributing to our clinical findings.

While we note that the strengths of this study include its relatively large size for a rare disease, availability of detailed medical records and questionnaire data, comprehensive laboratory evaluations, and use of a matched control patient cohort, we recognize that the sample size remains small which limits the power for robust statistical associations. We were also unable to conduct a systematic radiologic review of images and instead relied on medical record reporting. Since the patients were treated at different centers over different time periods, full ascertainment of medications was also limited. However, it remains notable that approximately one in five patients in our cohort was impacted by AVN and/or MTF.

Our findings highlight that AVN and MTF occur at a higher‐than‐expected frequency in patients with TBDs, with nearly half of patients experiencing their first event in childhood. In addition to vitamin D supplementation, DEXA scans and laboratory assessment of bone health at diagnosis and approximately annually thereafter are recommended for TBDs. Clinical trials are needed to determine whether bisphosphonate therapy may be effective in children and adults with TBDs and low BMD. Multi‐institutional clinical and basic science collaborations on AVN, MTF, and bone health are required to determine the optimum clinical management of these manifestations for patients with TBDs.

## Author Contributions

Study conception and design: N.G. and S.A.S. Data collection: A.M.N. and N.G. Data analysis: A.M.N., N.G., M.R.N., and S.A.S. Draft, revise, and approve final manuscript: A.M.N., N.G., M.R.N., and S.A.S.

## Ethics Statement

Individuals in this study were enrolled in the National Cancer Institute's Institutional Review Board‐approved Inherited Bone Marrow Failure Syndromes protocol (ClinicalTrials.gov identifier: NCT00027274). All study participants or their guardians signed written informed consent.

## Conflicts of Interest

The authors declare no conflicts of interest.

## Supporting information


**Data S1:** cge70038‐sup‐0001‐Supinfo.docx.

## Data Availability

There are restrictions to the availability of data from ClinicalTrials.gov identifier: NCT00027274 due to the importance of maintaining participant confidentiality. De‐identified data from that study may be requested from the corresponding author, Dr. Sharon A. Savage, and will be made available to qualified scientists after completion of appropriate data transfer agreements and NIH Institutional Review Board approval.
