# Supplementary material for: Avascular Necrosis and Minimal Trauma Fractures in Telomere Biology Disorders
Source: Clin Genet. 2025 Aug 5;109(2):286–93. doi: 10.1111/cge.70038 (PMC12779249; doi:10.1111/cge.70038)
Supplement: Supplementary file 1 — Data S1: cge70038‐sup‐0001‐Supinfo.docx. [file CGE-109-286-s001.docx]

**SUPPLEMENTARY INFORMATION**

**Supplementary Figure 1: Study overview.**

Abbreviations: AVN, avascular necrosis; DC, dyskeratosis congenita; MTF, minimal trauma fracture; n, number of participants.

**
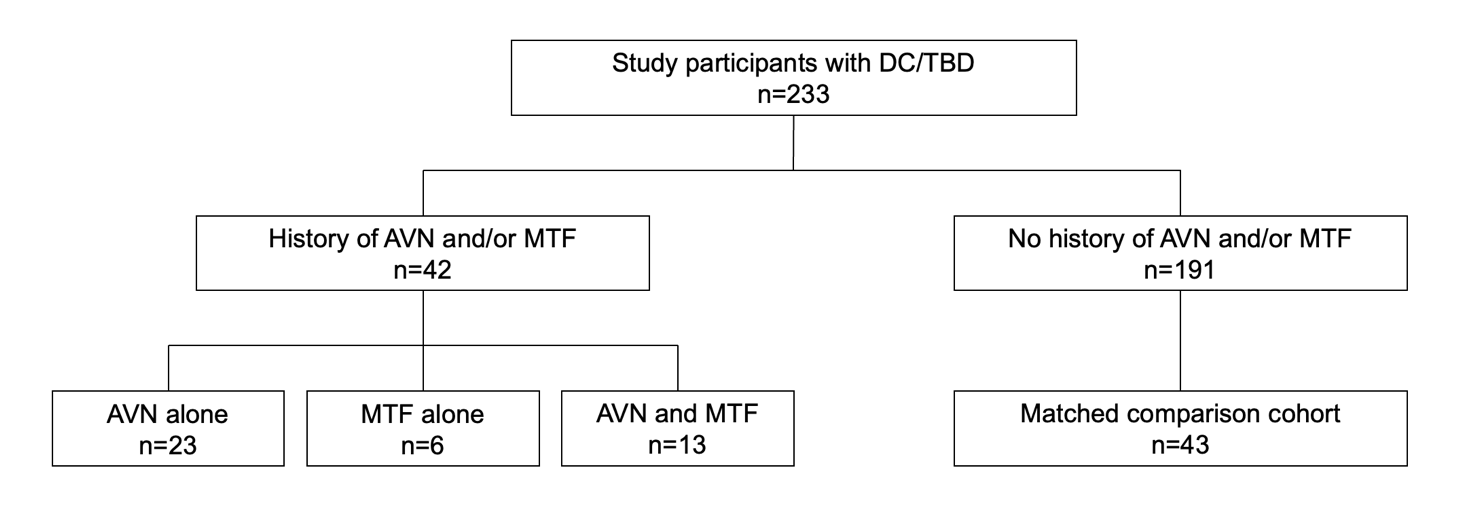
**

**Supplementary Table 1: Details of patients with avascular necrosis and/or minimal trauma fracture.** Bilateral events occurred in the same patient.

Abbreviations: AVN, avascular necrosis; HCT, hematopoietic cell transplant; MTF, minimal trauma fracture; NA, not applicable.

|  | # participants | # prior to HCT | Location | | # Events | # Bilateral Events | Associated gene |
| --- | --- | --- | --- | --- | --- | --- | --- |
| AVN only | 23 | 20 | Femoral head | | 22 | 15 | 2 *DKC1*; 1 *PARN* (AR); 2 *RTEL1* (AD); 8 *TERT* (AD), 1 *TERT* (AR); 3 *TERC* (AD); 3 *TINF2*; 3 unknown |
|  |  |  | Humoral head | | 3 | 1 |  |
|  |  |  | Knee | | 2 | 0 |  |
|  |  |  | Wrist | | 2 | 0 |  |
| MTF only | 6 | 4 | Femur | | 3 | 1 | 1 *CTC1* (AR); 1 *DKC1*; 1 *PARN* (AR); 2 *TINF2*; 1 unknown |
|  |  |  | Humerus | | 2 | 1 |  |
|  |  |  | Radius | | 1 | 0 |  |
|  |  |  | Ulna | | 1 | 0 |  |
|  |  |  | Tibia | | 1 | 0 |  |
|  |  |  | Vertebra | | 1 | NA |  |
| AVN and MTF | 13 | 8 | AVN | Femoral head | 11 | 7 | 3 *CTC1* (AR); 4 *DKC1*; 3 *RTEL1* (AR); 3 *TINF2*; 1 *WRAP53* (AR) |
|  |  |  |  | Humoral head | 2 | 1 |  |
|  |  |  |  | Knee | 1 | 1 |  |
|  |  |  |  | Vertebra | 1 | NA |  |
|  |  |  | MTF | Femur | 5 | 2 |  |
|  |  |  |  | Humerus | 4 |  |  |
|  |  |  |  | Clavicle | 3 | 1 |  |
|  |  |  |  | Tibia | 3 | 0 |  |
|  |  |  |  | Calcaneus | 2 | 0 |  |
|  |  |  |  | Radius | 1 | 0 |  |
|  |  |  |  | Wrist | 1 | 0 |  |
|  |  |  |  | 1^st^ digit | 1 | 0 |  |
|  |  |  |  | Vertebra | 1 | NA |  |

**Supplementary Table 2: Clinical parameters related to bone health in study participants.** No statistically significant differences were identified between affected groups and the matched patient cohort.

|  | AVN  N=36 | MTF  N=19 | AVN and/or MTF  N=42 | Matched Patient Cohort  N=43 |
| --- | --- | --- | --- | --- |
| Advanced Bone Age | 1 | 1 | 2 | 2 |
| Median Body Mass Index (range) | 22.3 (13.3-42) | 21.25 (13.3-25.8) | 22.3 (13.3-42) | 21.4 (14.5-36) |
| Median serum 25(OH)D (range) | 27 (13-74) | 26 (7-50) | 26 (7-74) | 23 (4-41) |
| Median serum calcium (range) | 9 (8.1-9.8) | 9.2 (8.1-10.2) | 9.1 (8.1-10.20) |  |
| Median serum creatinine (range) | 0.8 (0.4-2.3) | 0.7 (0.5-1.2) | 0.7 (0.4-2.3) |  |
| Median serum phosphorous (range) | 3.8 (1.9-6.3) | 3.9 (0.9-6.3) | 3.8 (0.9-6.3) |  |
| Median serum parathyroid hormone (range) | 37 (23-94) | 27 (15-55) | 37 (15-94) |  |

**Supplementary Table 3: Clinical features of study participants**

|  | AVN  N=36 | MTF  N=19 | AVN and/or MTF  N=42 | Matched Patient Cohort  N=43 | Entire Cohort  N=233 |
| --- | --- | --- | --- | --- | --- |
| Severe BMF (%) | 26 (72.2) * | 17 (89.5) * | 32 (76.2) | 19 (44.2) | 113 (48.5) |
| HCT (%) | 14 (38.9) | 12 (63.2) * | 20 (47.6) | 13 (30.2) | 70 (30.0) |
| Pulmonary Fibrosis (%) | 10 (27.8) | 8 (42.1) | 14 (33.3)*^,^ ** | 6 (14) | 46 (19.7) |
| PAVM (%) | 6 (16.7) | 7 (36.8) | 9 (21.4) )*^,^ ** | 0 | 13 (5.6) |
| Hepatopulmonary Syndrome (%) | 2 (5.6) | 4 (21.1) | 5 (11.9) )*^,^ ** | 0 | 6 (2.6) |
| GI Telangiectasia (%) | 6 (16.7) | 6 (31.6) | 9 (21.4) ** | 4 (9.3) | 17 (7.3) |
| Severe Liver Disease (%) | 5 (13.9) | 7 (36.8) | 9 (21.4) ** | 5 (11.6) | 22 (9.4) |
| Vascular Disease (%)^#^ | 10 (27.8) | 10 (52.6) | 14 (33.3) )*^,^ ** | 4 (9.3) | 26 (11.2) |
| DVT history or increased risk of thrombosis (%) | 8 (22) | 3 (15) | 9 (21) | 0 |  |
| Median LDL (range), N  Androgen  No Androgen | 147 (73-291), 12  122 (89-148), 7 | 145 (67-215), 6  93 (78-148), 5 | 125 (67-291), 15  95.5 (78-144), 10 | 102 (63-128), 6  88 (46 – 165), 26 |  |
| Median HDL (range), N  Androgen  No Androgen | 31 (11-160), 12  47 (8-87), 7 | 22 (11-57), 6  54 (23-72), 5 | 34 (11-160), 15  51 (8-87), 10 | 45 (10-51), 6  59 (41-121), 26 |  |
| Median Triglyceride (range), N  Androgen  No Androgen | 153 (58-264), 14  71 (39-125), 7 | 95 (48-264), 7  55 (38-74), 5 | 138 (48-264), 17  66 (38-125), 10 | 71 (42-180), 6  75 (30-158), 27 |  |
| Median Total Cholesterol (range), N  Androgen  No Androgen | 202 (150-393), 14  186 (148-204), 7 | 183 (120-243), 7  169 (142-186), 5 | 191 (120-393), 17  171 (142-204), 10 | 146 (114-199), 6  162 (102-258), 27 |  |
| Statin Use, N | 2 | 1 | 2 | 1 |  |

^#^Includes patients with clinically significant pulmonary arteriovenous malformations and/or gastrointestinal telangiectasias.

*Denotes a statistically significant. difference between AVN and/or MTF+ compared to the Matched Cohort with a p-value < 0.05

**Denotes a statistically significant. difference between AVN and/or MTF+ and the Entire Cohort with a p-value < 0.05
